# Beneficial effects of endophytic fungi inoculation on tanshinones and phenolic compounds of *Salvia abrotanoides*

**DOI:** 10.22038/IJBMS.2023.67730.14828

**Published:** 2023-04

**Authors:** Fatemeh Masoudi Khorasani, Ali Ganjeali, Javad Asili, Monireh Cheniany

**Affiliations:** 1 Department of Biology, Faculty of Science, Ferdowsi University of Mashhad, Mashhad, Iran; 2 Department of Pharmacognosy, School of Pharmacy, Mashhad University of Medical Sciences, Mashhad, Iran

**Keywords:** Cryptotanshinone, Endophytic fungi, Penicillium canescens, Salvia abrotanoides, Talaromyces sp., Tanshinone IIA

## Abstract

**Objective(s):**

*Salvia abrotanoides* is considered as a new source of tanshinone-producing plants in Iran. Symbiosis of endophytic fungi with their host plants is an effective tool to promote the growth and secondary metabolism of medicinal herbs. Therefore, using endophytic fungi as a biotic elicitor is a proper solution to increase the yield of plant products.

**Materials and Methods:**

In this study, some endophytic fungi were first isolated from the root of *S. abrotanoides*, then two of them (*Penicillium canescens* and *Talaromyces *sp.) were co-cultivated with the sterile seedling of *S. abrotanoides* in pot culture. After proving the colonization of these fungi in the root tissues by microscopic studies, their effects on the production of critical medicinal compounds such as tanshinones and phenolic acids were investigated in the vegetation stage (120 days).

**Results:**

Our results showed that the content of cryptotanshinone (Cry) and tanshinone IIA (T-IIA) in plants inoculated with *P. canescens* increased by 77.00% and 19.64%, respectively, compared with non-inoculated plants (control). The contents of mentioned compounds in plants inoculated with *Talaromyces *sp*.* increased by 50.00% and 23.00%, respectively. In this case, in plants inoculated with *P. canescens*, it was found that the level of caffeic acid, rosmarinic acid, and its PAL enzyme activity increased by 64.00%, 69.00%, and 50.00%, respectively, compared with the control.

**Conclusion:**

Endophytic fungi have specific modes of action and the ability to provide multiple benefits. Each of the two strains is a highly considerable microbial resource for the growth and accumulation of active compounds of *S. abrotanoides.*

## Introduction

Endophytic fungi positively affect their host plants, including secondary metabolite production, growth promotion, and disease-resistance improvement. Therefore, it is a stimulating and stable strategy to use endophytic fungi for the quality enhancement of medicinal herbs or important crops (1). Considering the limitations associated with the productivity and vulnerability of plants, fungal endophytes could be used as alternative or more efficient elicitors compared with other biotic and abiotic elicitation methods (2). Plant-microbial symbiotic systems are essential for plants. Plant growth-promoting fungi (PGPF) that increase yield and accumulate secondary metabolites are beneficial for medicinal plants. *Piriformospora Indica* has been used to increase asiaticoside production in seed tissue culture of *Centella asiatica* (3) and fungus *Mycena sp*. F-23 has grown contents of ginsenosides and flavonoids *Anoectochilus formosanus* in pot culture (4). 

Lamiaceae species are potential sources of medicinal compounds and natural antioxidants due to their high content of polyphenols and terpenoids. The plant Brazambel (Persian name for *S. abrotanoids*) is distributed in the northern, eastern, and central regions of Iran and grows in mountainous areas with cold and semi-arid climates. It is a shrub or semi-shrub plant with a height from 1 to 1.5 meters propagated by seeds (5). It has been reported that tanshinones, abietane-type norditerpenoid quinones, are the most abundant and vital bioactive compounds in the roots of this species for their medicinal application (6-8). This plant is a cooling medicine to treat fever (9). Also, it has antimicrobial, anticancer, and antioxidant activities and is used in treating cardiovascular diseases, liver fibrosis, and cough (10-12). The phenolic and terpene compounds of the stems and leaves of this plant have anti-leishmaniasis properties (13). Traditional methods of tanshinones production are inadequate to meet the rapidly rising demand because yield levels are low, and the plants are very slow growing. Therefore, using PGPF as biofertilizers in medicinal plants is promising and has benefits that include improving the quantity and quality of crops and reducing pollution in different ecosystems (14). 

This study addresses the inoculation effects of two endophytic fungi (*Talaromyces *sp., *P. canescens*) associated with *S. abrotanoides* on critical medicinal compounds derived from the same plant in greenhouse conditions in the vegetation phase.

## Materials and Methods

***Plant sampling and isolation of fungal endophytes from the roots of S. abrotanoides***

**Figure 1:**
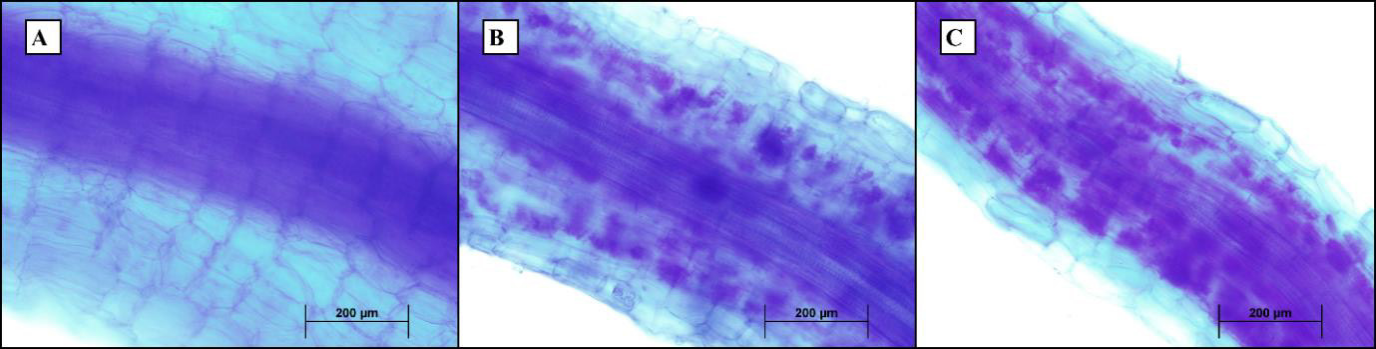
Longitudinal section of *Salvia abrotanoides* root inoculated with endophytic fungi. (A) Control sample; (B) Intense colonization of *Talaromyces *sp*.* (formation of arbuscules); (C) Intense colonization of *Penicillium canescens *(formation of arbuscules)

**Figure 2:**
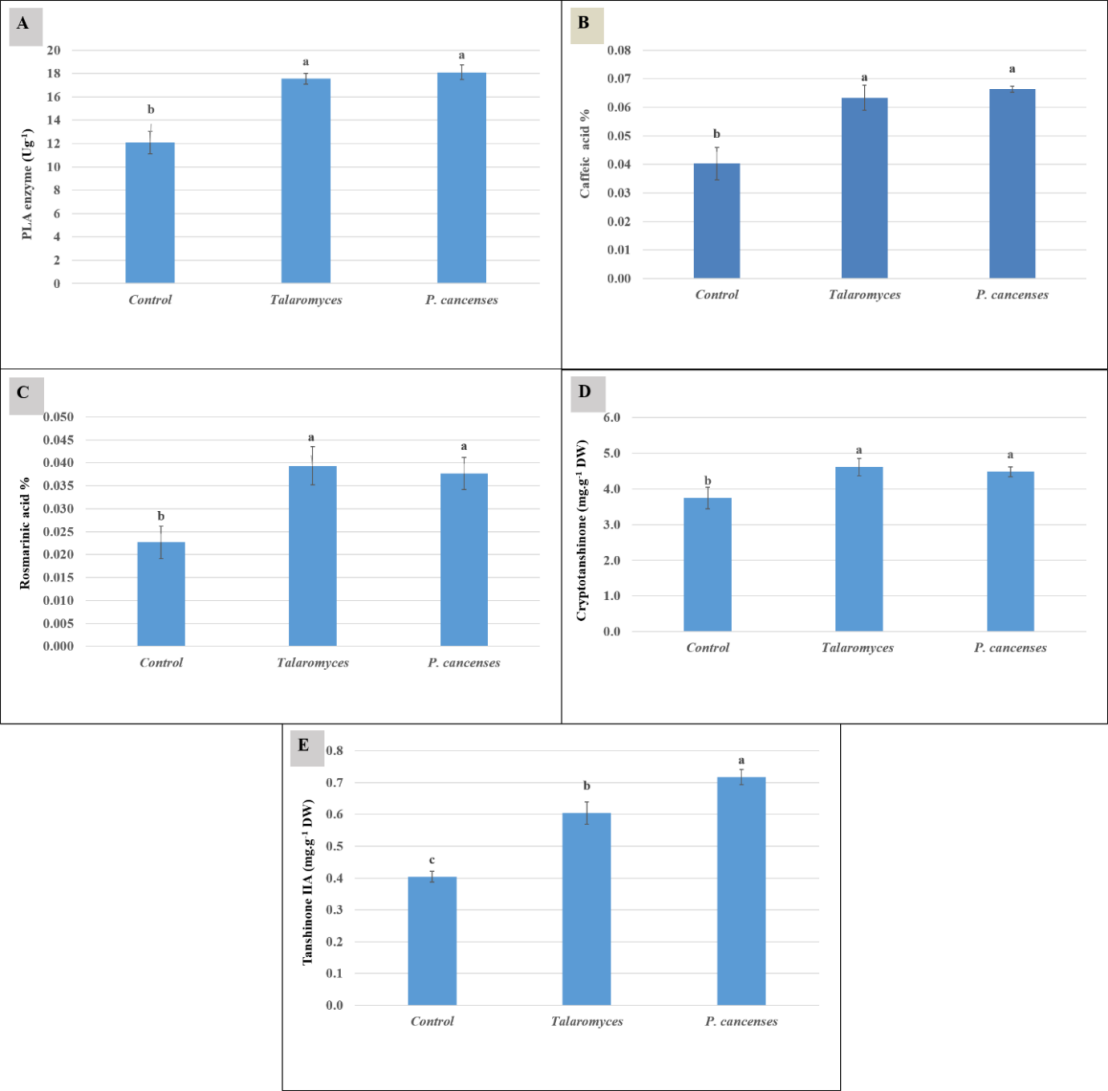
Effects of fungal inoculation on secondary metabolite production of *Salvia abrotanoides*. PAL enzyme activity (A), caffeic acid (B), rosmarinic acid (C), cryptotanshinone (D), and tanshinone IIA (E) were shown. Error bars show the SE between biological replicates, and Duncan’s test was performed between different treatment groups. Other letters (a, b, and c) indicated significant differences (*P<*0.01) between samples

The roots of *S. abrotanoides* were collected from the Zoshk area (N 36^°^16ʹ58ʺ, E 59^°^07ʹ06ʺ) near Mashhad, *Khorasan Razavi province,* northeast of Iran, in the flowering stage, and voucher specimens were deposited at the herbarium of Ferdowsi University of Mashhad *(FUMH)*, Iran, under number 36763. The plant material was kept at 4 ^°^C, and fungal isolation was fulfilled 24 hr after sampling. Surface sterilization of the roots was performed according to the method of Fisher *et al*. (15). For this goal, the roots of *S. abrotanoides* were thoroughly washed through running water. After several rinses with distilled water, they were immersed in 70% ethanol solution, 2.5% sodium hypochlorite, and 70% ethanol solution for 1 min, 3 min, and 30 sec, respectively. At the end of each step, the samples were washed four times with sterile distilled water and dried with sterile filter paper. The roots were then cut into 1 cm pieces using a sterile knife and placed on sterilized Petri dishes containing 30 ml of malt extract agar (MEA) plus 100 mg/l streptomycin to avoid bacterial contamination. To prove the removal of epiphytic microorganisms, 0.3 cc of distilled water from the last washing step was placed on MEA as a control. The absence of microbial growth on the culture medium confirmed the surface sterilization method. The Petri dishes were sealed with Parafilm and incubated at 25±2 ^°^C until fungal colonies emerged from the root fragments (Figures 1 and 2). After the appearance of the fungal colony, the tip of each colony’s hyphae was subcultured in a new petri dish containing streptomycin-free malt extract agar medium to obtain the pure fungal isolates. The endophytic fungi and metabolic profile of each were identified by molecular approach and LC-mass (16). 

***Endophytic fungi preparation for inoculation***

After identifying the metabolic profile of each fungus isolated from *S. abrotanoides*, two fungi, *P. canescens* and *Talaromyces *sp.*,* were selected based on their potential to produce tanshinones by themselves. Therefore, the first one was the producer of the highest amount of cryptotanchinone, and the second one was not the producer of this compound. They were used for the elicitation of *S. abrotanoides* plants. Each strain was separately cultured in a solid culture of MEA to create a single active colony. Then, a fungal mycelial agar plug with a diameter of 4 mm was removed from the tangible culture medium to produce sufficient mycelial mass and transferred to a liquid culture medium (100 ml Potato Dextrose Broth (PDB) in 250 ml Erlenmeyer flasks, incubated on a shaking incubator at 30 ^°^C with 100 rpm for 14 days) to reproduce the fungus. Filtration of the culture medium was done with Whatman filter paper No. 1, and homogenization was carried out by centrifugation at 12000 rpm for 5 min. At least 10 ml of each suspension was mixed with 10 g of sterilized soil and used as an inoculum (17).

***Preparation of sterile plants***

Seeds of S*. abrotanoides* plants were collected in the Zoshk area (already mentioned). Surface sterilization of seeds was conducted by brief washing with soap liquid, immersion in 70% ethanol for 30 sec, rinsing with distilled water, and immersion in 2% sodium hypochlorite for 7-8 min. For the last part of the sterilization, seeds were washed 4 times with sterile distilled water (each time for 5 min). Then, they were placed on wet filter paper in a sterile petri dish. The Petri dishes were transferred to a dark culture chamber at 22±1 ^°^C for germination. Germinated seeds were exposed to light to make seedlings 2-3 cm long.

***Inoculation of sterile seedling S. abrotanoides by fungal mycelia***

Sterile seedlings were planted in 2 kg pots filled with soil and sand (1:1 ratio). The soil was previously disinfected by autoclave at 121 ^°^C and 1 atmospheric pressure for 30 min. For fungal inoculation, 10 ml of each suspension mixed with 10 g of sterilized soil was added around the roots. In each pot, 10 seedlings were planted at a depth of 2 cm. Finally, 5 plants were selected and maintained in a controlled temperature condition of 22±1 ^°^C, 16/8 hr light/dark respectively, and 60% humidity. The surface of the pots was covered with a plastic layer for 10 days to retain humidity. During the first 10 days, the pots were irrigated with sterile distilled water. After that, irrigation was done twice a week for 45 days with 0.5 M Hoagland and finally with 1 Hoagland solution. The pots were placed under the net rooms in the greenhouse of the Faculty of Agriculture, Ferdowsi University of Mashhad, Iran. The pots were brought out of the net room two months later to grow in normal condition. At the end of the experiment, 120 days old plants (vegetation stage) were collected and divided into two parts: roots and shoot, and then dried in the shade.

***The percentage of colonization***

After removing and washing the roots with the distilled water, they were put in fixator FAA (formalin, acetic acid, and 50% ethanol by a ratio of 5:5:90). The one hundred 1cm root pieces were selected to determine the percentage of colonization. The pieces were placed in 10% potassium hydroxide at room temperature for 24 hr, then washed with distilled water and kept in 10% hydrochloric acid for 3 min to soften the tissues. Finally, they were stained with 0.05% trepan blue in Lacto phenol (18). The presence of fungal structures such as mycelium, spores, vesicles, and arbuscules in root parts was examined with a 40x magnification of a Nikon microscope equipped with a camera (CCD). The colonization percentage was calculated by the Trouvelot method and MYCO CALC software (19).

***Phenylalanine ammonia-lyase (PAL) activity***

For the extraction, 0.3 g of the fresh leaf was ground in 6.5 ml extraction buffer (50 mM tris chloride buffer with 8.8 acidities and 15 mM beta-mercaptoethanol) and homogenized using a cold porcelain mortar. The extract was centrifuged at 12000 rpm for 30 min at 4 ^°^C. After centrifugation, the above solution was used for measurements. The reaction mixture with a final volume of 2 ml was prepared, including 1 ml Tris-HCL buffer, 0.5 ml of 10 M phenylalanine, 0.4 ml distilled water, and 0.1 ml plant extract. The reaction mixture tube was placed in the water bath at 37 ^°^C for 1 hour; then, the reaction was stopped by adding 0.5 ml of 6 M acetic acid. For the next step, 15 ml ethyl acetate was added to the tube and composed of two phases. The oily phase was separated, leaving the residue under the laminar hood at room temperature to evaporate. Finally, the precipitate, which is cinnamic acid, was dissolved in 3 ml of 0.05 M NaOH (20). The absorbance of each sample was recorded at 290 nm, and a standard curve measured cinnamic acid concentration. Enzyme activity was calculated based on the conversion rate of phenylalanine to trans-cinnamic acid. One unit of PAL activity is equivalent to one micromole of cinnamic acid produced per minute.

***HPLC analysis***

Rosmarinic (RA) and caffeic acids (CA) were measured by the high-performance liquid chromatography (HPLC) method. The shoot parts of *S. abrotanoides* were dried at room temperature and shade to prepare the extracts for phenolic acid evaluation. For extraction, 0.5 g of shoot parts powder was mixed with 25 ml of 70% methanol, and the tubes were placed in an ultrasonic device at 30 ^°^C for 20 min. The extracts were filtered using Whatman No. 1 filter paper and used for HPLC analysis (21). Identification and measurement of RA and CA were carried out by an HPLC device (Knauer, Germany), equipped with a K-1001 pump, a K-2600 UV detector, and analytical column C8 (particle size 5 μm, 250×4.6 mm) at 22 ^°^C. The injection volume and flow rates were 20 μl and 0.8 ml/min, respectively. The mobile phase for the analysis of plants’ extracts was a mixture of deionized water (A) and methanol (B) in the following gradient elution program: 0-14 min: 0-90% A, 14-16 min: 0-0% A, 16-22 min: 0-90% A, 22-24 min: 90-90% A. Quantitative measurements of the samples were conducted based on standard curves of CA and RA absorbance at 320 nm (maximum absorbance of CA and RA). Data processing was performed by EZChrom Elite software (22).

***Determination of tanshinone IIA and cryptotanshinone content by NMR method***

The roots of *S. abrotanoides* were dried at room temperature and shade and then powdered. The ultrasonic method was used to extract the samples. For this, 0.5 g of root powder was mixed with 5 ml of pure ethyl acetate and placed in ultrasonic bath water (Parasonic 2600S, Japan) for 30 min at 30 ^°^C (4 times). The extracts were filtered through a 0.45 µm syringe filter. After 70 mg of each extract were vortexed with 2.0 mg of DMSO_2_ in DMSO- d6 (0.4 ml), and 360 μl of each sample was transferred to a 5 mm NMR tube; Deuterated chloroform (CDCl_3_) was also used as the sample solvent. One-dimensional H-NMR spectra using NMR apparatus 600 MHz Bruker were applied to determine the concentrations of T- IIA and Cry.

Dimethyl sulfone (DMSO_2_, purity 98 %) and DMSO-d6 were provided from Sigma (St. Louis, MO, USA). ^1^H-NMR spectra were recorded in DMSO-d6 at 298 K with the 5 mm NMR probe. ^1^H-NMR spectra were recorded using the following parameters: spectra width; 6009.6 Hz, acquisition time; 5.4 sec, free induction decay (FID) data points; 64 K relaxation delay; 3.0 sec, and flip angle; 30^°^ were applied before Fourier transformation.

The values were measured by comparing the signal areas of selected protons of each compound to be quantified with those of DMSO_2_. The areas were measured with Mnova (version 14.0.0- 23239; Mestrelab Research S.L) software. The baseline and phase were corrected manually.

Based on the current results, the successful application of qH NMR requires that at least one non-overlapping signal be quantified for each analyte. In this study, DMSO_2_ was chosen as an internal standard with a strong H singlet (6 H) in ^1^H NMR spectra of the extract (around 3.02 ppm). After integration of the ^1^H NMR spectrum, the contents (mg) of cryptotanshinone, tanshinone IIA, and internal standard (IS) were calculated using the following equation:

Mx = Ix/Ist × Mst/Mwst × Nst/Nx × Mwx

Where I: is the integral area of DMSO_2_ (3.02, S), cryptotanshinone (4.89, T), and tanshinone IIA (7.23, brS), N: is the number of protons for DMSO_2_ (6 H), cryptotanshinone (1 H) and tanshinone IIA (1 H), Mw: is the molecular weight of cryptotanshinone: 296, DMSO_2_: 94, and tanshinone IIA: 294, Mx: is the content of cryptotanshinone and tanshinone IIA, Mst: is the content of internal standard (IS=2 mg). 

***Statistical analysis***

Statistical analyses (ANOVA) were performed using SPSS software version 25.0.0 to test the significant differences between inoculation and some fungi. The experiment was set up in a completely randomized design with three replications. Duncan’s test (*P*<0.01) was used to compare the mean value of each treatment group.

## Results

***Fungal Colonization***

The percentage of fungal colonization varied between 71.00% and 53.00% among *P. canescens*, *Talaromyces *sp. (Table 1). The highest percentage of endophyte colonization was related to *P. canescens* treatment (71.00%). No colonization was observed in control. The longitudinal section of roots inoculated with endophytic fungi showed the intense colonization of *P. canescens* and the penetration of *Talaromyces *sp. Hyphae in epidermal cells (Figure 1).

The results of the analysis of variance at the vegetation stage showed that inoculation of *S. abrotanoides* by fungal species had significant effects (*P*<0.01) on the production of secondary plant metabolites (Table 2.).

***PAL enzyme activity and phenolic acids content***

Our results showed that PAL enzyme activity was enhanced in plants inoculated by *Talaromyces *sp. (17.55 Ug^-1^) and *P. canescens* (18.11 Ug^-1^), respectively, and its activity was almost 1.64 times more than the control. Also, the treatments had no significant difference (*P*<0.01) from each other for increasing the PAL enzyme activity of the plants (Figure 2A).

**Figure 3:**
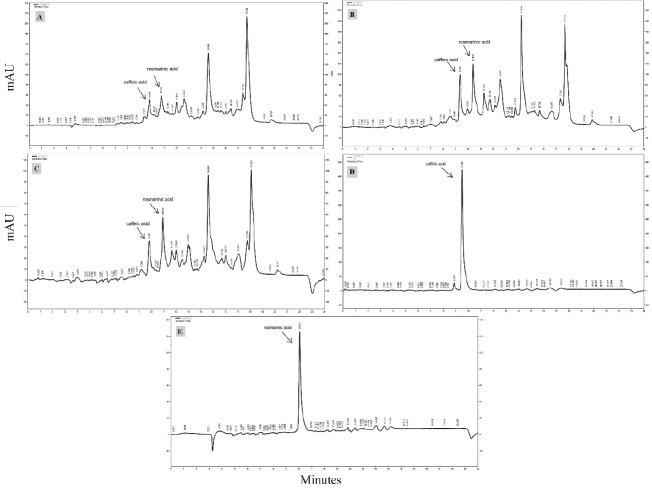
HPLC chromatograms of *Salvia* *abrotanoides’* shoot extract and standards. (A) control shows CA and RA of *S. abrotanoides’* shoot extract non-inoculated with retention times 9.783 and 10.900, respectively; (B) shows CA and RA of *S. abrotanoides’* shoot extract inoculated by *Talaromyces *sp*.* with retention times 9.360 and 10.400 respectively; (C) shows CA and RA of *S. abrotanoides’* shoot extract inoculated by *P. canescens* with retention times 9.667 and 10.883, respectively; (D) shows a standard chromatogram of CA, Retention time is 9.500 min; (E) shows a standard chromatogram of RA, Retention time is 10.067 min

The HPLC method measured phenolic acid content, including CA and RA (Figure 3). Our results showed that the content of CA in plants inoculated by *Talaromyces *sp. (0.06 mg/g DW) and *P. canescens *(0.07 mg/g DW) was 56.33% and 64.00% more than the control. A similar trend was observed in the case of RA. The RA content in plants inoculated by *Talaromyces *sp. (0.039mg/g DW) and *P. canescens *(0.037mg/g DW) was 69.57 and 57.83% more than the control, respectively; it should be mentioned that neither fungus species had any significant difference in improving the plants’ CA and RA content (Figures 2B , 2C).

***Tanshinones content***

**Figure 4:**
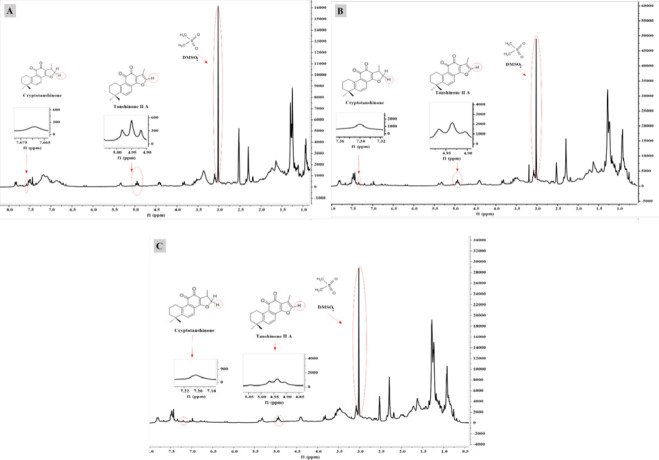
The ^1^H-NMR spectra of cryptotanshinone and tanshinone IIA in Control (A), *Talaromyces *sp*. *(B), and *Penicillium canescens* (C). These compounds were quantified using distinguishing signals of H-16 for cryptotanshinone, tanshinone IIA, and DMSO2

The H-NMR method qualified and quantified T-IIA and Cry (Figure 4). The results of H-NMR detection showed that Cry content in plants inoculated by *Talaromyces *sp. (4.48 mg/g DW) and *P. canescens (*4.60 mg/g DW) was 23.14% and 19.64 % more than the control. Fungus species had no significant difference (*P*<0.01) with each other for increasing the Cry content of the plants (Figure 2D). The highest content of T-IIA was observed in plants inoculated by *P. canescens* (0.72 mg/g DW) that increased 77.00% compared with the control. The content of T- IIA in plants inoculated by *Talaromyces *sp. (0.61 mg/g DW) was 50% more than the control (Figure 2E). 

## Discussion

Endophytes have been introduced as effective microbial and plant growth sources because they can help their host consistently and sustainably under a wide range of ecological conditions (23). Chemical fertilizers may replace endophytic fungi to increase the productivity of some medicinal or crop plants. These endophytes can be used as biofertilizers or disease prevention agents because they do not impose ecological restrictions and are compatible with the environment (24). Our results indicated successful inoculation of each of the two fungal endophytes on colonization of root cells. In this study, the highest rate of colonization and formation of fungal structures was observed in plants inoculated by *P. canescens* with a frequency of 71.00%. Studies have shown that the colonization process varies according to the type of plant and media, fungal strain, fungal life cycle and plant response to it, plant age at inoculation, and plant growth conditions (25). 

Variation in the expression of genes encoding key enzymes in a biosynthetic pathway directly affects the accumulation of the corresponding secondary metabolites. Phenolic acids are biosynthesized via the phenylpropanoid and tyrosine-derived pathways (26). As terpenoids, tanshinones are produced by both the mevalonate pathway (MVA) in the cytosol and the methylerythritol phosphate (MEP) pathway in plastids (27). Many key enzyme-encoding genes in *S. miltiorrhiza *have been identified, such as those for the PAL enzyme (28). PAL is the first key enzyme of the phenylpropanoid pathway, and its expression is closely related to the accumulation of lignin, anthocyanin, and other phenolic compounds (29). Our results showed that both endophytic fungi significantly increased PAL enzyme activity in inoculated plants. This result was attractively consistent with the increase in CA and RA in the shooting part of *S. abrotanoides*. In the same context, other studies have shown that the expression of PAL was associated with the synthesis of RA and salvianolic acid in *S. miltiorrhiza* in the presence of various stimuli (e.g., abscisic acid, fungal extracts, and folic acid) (30, 31). Also, it was reported that *Alternaria sp*. A13 promoted the accumulation of practical components in the roots of *S. miltiorrhiza*, with increases in the total phenolic acid and lithospheric acids A and B contents of 210, 128, and 213%, respectively (32). Our results showed that the biotic elicitors significantly increased the synthesis of Cry and T-IIA compared with the control. The highest amount of Cry belonged to plants inoculated with *P. canescens*. These findings align with other studies on using bio-elicitors for increasing tanshinones. For example, in one study, the effects of elicitation of *S. miltiorrhiza* by a strain of *C. globosum* D38 and its mycelial extract increased the synthesis of salvianolic acids and tanshinones, especially dihydrotanshinoneI and Cry increased due to multiplying the transcription of essential genes in the tanshinone biosynthetic pathway significantly (33). The endophytic fungus *Phoma herbarum* D603, isolated from *S. miltiorrhiza*, stimulated root growth and development by producing auxin hormone and siderophores and improved plant nutrition by solubilizing phosphorus. In addition, it causes the accumulation of tanshinones by regulating the expression level of key genes of the tanshinone synthesis pathway (34).

**Table 1:** Means comparasion for fungal colonization percentage after eight weeks from inoculation

	**%F**	**%m**	**%a**	**%A**
***P. canescens***	71±0.5^b^	43.79±0.5^b^	55.86±0.5^b^	17.37±0.5^b^
***Talaromyces sp.***	53±0.5^c^	43.94±0.5^b^	24.12±0.5^c^	5.62±0.05^c^
**Control**	0	0	0	0

Frequency of inoculation F (%), the intensity of colonization within individual roots m (%), arbuscular richness in root fragments where the arbuscules were present a (%), and arbuscular abundance in the root system A (%)

**Table 2:** Analysis of variance for physiological parameters of *Salvia abrotanoides* in vegetation stage

S.O.V	Df	Mean Square				
		Cry	T- IIA	CA	RA	PAL
Elicitor	2	0.655^**^	0.075^**^	0.001^**^	0.00001^**^	33.181^**^
Error	6	0.058	0.001	0.00001	0.00001	0.515
					

*, **: Significant at the 0.05 and 0.01 probability levels, respectively

## Conclusion

This study investigated the effects of the inoculation of two endophytic fungi as biotic elicitors on the content of specific metabolites, including Cry and T-IIA in *S. abrotanoides*. Our results showed that infection of the plants by an endophytic fungus (*P. canescens* or *Talaromyces *sp.) effectively increased the production of mentioned compounds and phenolic acids. Our results revealed a positive correlation between PAL activity and the content of phenolic acids in plants inoculated by *P. canescens* and *Talaromysces sp*. Meanwhile, the accumulation of tanshinones, especially Cry and T-IIA, was more than CA and RA. Therefore, both isolates of *P. canescens* and *Talaromyces *sp*.* are highly beneficial microbial resources, especially *P. canescens*, which can be used to enhance the yield and quality of metabolites* S. abrotanoides* in medicine.

## Authors’ Contributions

FMK, AG, JA, and MC conceived the study and design; FMK, AG, and JA helped with data acquisition; FMK, AG, and JA analyzed and interpreted data; FMK, AG, and JA wrote the original draft; FMK, AG, and JA reviewed and edited the manuscript; FMK, AG, JA, and MC provided supervision. All authors have read and agreed to the published version of the manuscript. 

## Conflicts of Interest

The authors declare that they have no competing interests. 
